# Potential of Native Arbuscular Mycorrhizal Fungi, Rhizobia, and/or Green Compost as Alfalfa (*Medicago sativa*) Enhancers under Salinity

**DOI:** 10.3390/microorganisms8111695

**Published:** 2020-10-30

**Authors:** Raja Ben-Laouane, Marouane Baslam, Mohamed Ait-El-Mokhtar, Mohamed Anli, Abderrahim Boutasknit, Youssef Ait-Rahou, Salma Toubali, Toshiaki Mitsui, Khalid Oufdou, Said Wahbi, Abdelilah Meddich

**Affiliations:** 1 Laboratory of Agro-Food, Biotechnologies and Valorization of Plant Bioresources, Department of Biology, Faculty of Science–Semlalia, Cadi Ayyad University, BP 2390, 40000 Marrakesh, Morocco; benlaouaneraja@gmail.com (R.B.-L.); mohamed.aitelmokhtar@gmail.com (M.A.-E.-M.); moh1992anli@gmail.com (M.A.); Abderrahim.boutasknit@gmail.com (A.B.); youssefaitrahou41@gmail.com (Y.A.-R.); salma.toubali@edu.uca.ac.ma (S.T.); wahbi@uca.ma (S.W.); 2 Laboratory of Biochemistry, Faculty of Agriculture, Niigata University, Niigata 950-2181, Japan; t.mitsui@agr.niigata-u.ac.jp; 3 Laboratory of Microbial Biotechnologies, Agrosciences, and Environment, Department of Biology, Faculty of Science Semlalia, Cadi Ayyad University, BP 2390, 40000 Marrakesh, Morocco; oufdou@uca.ac.ma

**Keywords:** Antioxidant status, autochthonous biofertilizers, by-product, microbiome, nutrient uptake, salt-tolerance mechanisms

## Abstract

Salinity is one of the devastating abiotic stresses that cause reductions in agricultural production. The increased salinization affects alfalfa growth, metabolism, and rhizobium capacity for symbiotic N_2_ fixation negatively. This study was undertaken to investigate the efficiency of green compost (C; made from green waste), arbuscular mycorrhizal fungi (M; field-sourced native consortium), and/or rhizobium (R; a salt-tolerant rhizobium strain) individually or in combination as an effective strategy to improve alfalfa productivity under non-saline and high-saline (120 mM NaCl) conditions. In addition, we aimed to understand the agro-physiological and metabolic basis as well as glomalin content in the soil of biofertilizers-induced salt tolerance in alfalfa. Here, we show that mycorrhizal infection was enhanced after MR inoculation, while C application decreased it significantly. Salinity reduced growth, physiological functioning, and protein concentration, but the antioxidant system has been activated. Application of the selected biofertilizers, especially C alone or combined with M and/or R improved alfalfa tolerance. The tri-combination CMR mitigated the negative effects of high salinity by stimulating plant growth, roots and nodules dry matters, mineral uptake (P, N, and K), antioxidant system, synthesis of compatible solutes, and soil glomalin content, sustaining photosynthesis-related performance and decreasing Na^+^ and Cl^−^ accumulation, lipid peroxidation, H_2_O_2_ content, and electrolyte leakage.

## 1. Introduction

In the last decade, considerable attention has been paid to soil salinity due to its devastating effects on plant growth and development. It is estimated that approximately 8% of global land encounter salinity problems, which are increasing rapidly with an estimated yearly addition of 0.3–1.5 million ha of farmland, reducing crop production over than 20% [1]. These losses are of great concern mainly for the agriculture-based countries. Inefficient irrigation and the use of saline water on agricultural land have led to saline soils, mainly in arid and semi-arid regions. In addition, increasing industrialization and climate change exacerbate this situation [1,2]. Globally, the degree of salinization has economic and social implications and may hamper the food security and agricultural profits. Salinity affects plants in three critical ways: osmotic, ionic, and/or oxidative, which have a negative impact on crop growth and productivity [3]. Osmotic stress leads to an alteration of the water potential, reducing the efficiency of water use by the plant due to the induction of physiological drought conditions. Ionic stress disrupts mineral homeostasis at the cellular and whole plant level, while oxidative stress causes the release of reactive oxygen species (ROS), which inhibit cell growth and plant metabolism. The degree of salt tolerance varies significantly with plant species and cultivars, thereby differing in their capacity to remain productive as soil salinity increases.

Alfalfa (*Medicago sativa* L.) is one of the most agronomic and economic important forage crops worldwide, being more highly persistent than most common field crops [4]. The ecological interest in alfalfa relies on its ability to fix atmospheric nitrogen in symbiosis with rhizobia inside nodules, which allows the reduction or avoidance of N fertilizer application [4]. Due to its high biomass production, high nutritional quality, high protein content, and its perennial growth, alfalfa is becoming more reliable and a great potential species as feedstock for animal and as a sustainable crop for second-generation bioethanol production [5,6]. Alfalfa is widely cultivated in arid and semi-arid areas where salinity is one of the main factors limiting the growth of this crop [7,8], and it strongly contributes to the socio-economic development of the local community [4]. Alfalfa is considered as moderately tolerant to salt and shows a clear stunting and evident yield reduction when the salinity value approaches 20 mM NaCl [9].

Several strategies have been developed using conventional breeding, marker-assisted selection, and plant genetic engineering to release new salt-tolerant varieties and decrease the toxic effects caused by high salinity on plant fitness [3,10]. However, the overall progress of these techniques has been slow, and the identification of critical genetic determinants of stress tolerance is a challenging task. In addition, these practices are not always financially feasible for farmers. Plants are intimately associated with vast numbers of microorganisms in the rhizosphere that provide host functions change in response to stresses and environmental stimuli [11]. In particular, pioneering studies suggest that plants adopt the “cry-for-help” strategy by changing their root exudation chemistry to recruit beneficial microorganisms to resist abiotic and biotic stresses [12]. The approach based on the use of beneficial soil microorganisms such as arbuscular mycorrhizal fungi (AMF) and rhizobia could be considered as an effective strategy to salinity issues in sustainable agriculture programs. Both mycorrhizal and rhizobial symbioses are known to increase tolerance to abiotic stresses in the host plants by modulating diverse mechanisms and improving their growth and productivity. It has been reported that the co-inoculation and co-culture of microbes could be more beneficial and enhance plant growth than a single-strain inoculation [13]. AMF improve nutrient and water uptake, increase photosynthetic rate, and induce the antioxidant system to prevent ROS damages [14,15]. AMF could ameliorate soil physicochemical and biological properties mainly through glomalin, a glycoprotein substance, which has been shown to increase soil aggregate stability and soil water potential [16]. The symbiosis between *Rhizobium* species and legumes allows fixing the atmospheric N_2_, and it represents a renewable source of N for agriculture. This association is cheaper and usually more effective as an agronomic practice for ensuring an adequate supply of N for legume-based crop and pasture production than the application of N fertilizers, since it could satisfy 80 to 90% of the total plant N requirements [17]. In addition to this nutritional effect, these bacteria can alleviate the negative impacts of salt stress through mechanisms including acidification of the rhizosphere, enhancement of root surface area, increasing root exudates (i.e., exopolysaccharides), improving the leaf water status of the host plant, enhancing nutrient availability, and the release of bacterial volatiles as an inducer of systemic resistance [18]. The production of the enzyme ACC (1-aminocyclopropane-1-carboxylate) deaminase reduces the plant ethylene levels and thus facilitates plant growth under environmental stresses [19]. These microorganisms possess growth-promoting traits, including P and K solubilization, N fixation, specific enzymatic activity secretion, and the production of phytohormone and biofilm, thus permitting the improvement of plant growth and biomass.

The efficiency of the health-promoting soil microbiomes strategy can be promoted by the assistance of organic amendments to the soil such as green compost. This latter represents both an interesting agricultural practice for the recovery of depleted soils and a sustainable waste recycling management. The positive effects of compost-based biofertilizers on plant growth, yield, and stress tolerance have been reported in several crops [20,21]. Compost could enhance soil quality (water-holding capacity and enrichment of humus and nutrients) and microbial activities [17]. Furthermore, it plays a pivotal role in inducing salinity tolerance via supplying plants with ample nutrients for their cellular and structural needs [7] and stimulation of antioxidant system [20]. It is likely that in organic agricultural production systems, there was a positive interaction of the root-associated microorganisms, which may benefit the cultivation of the crops under normal and environmental stress conditions. However, how the composts exert positive effects on plant growth is still to be elucidated, and there remain knowledge gaps to determine if the application of compost influences rhizobia and AMF functions. Most of the studies have focused on AMF and/or rhizobia inoculation [8,9] or the application of compost [7] to alleviate the adverse impact of salt stress on alfalfa plants. However, to our knowledge, no data are available about the effect of their combined applications and the underlying salinity tolerance mechanisms in alfalfa. Therefore, it is crucial to improve the salt tolerance of alfalfa to combat increasing agricultural water-, earthquake-, and tsunami-induced soil salinity. In this study, we focused on microbiome engineering by evaluating the combined practices of native AMF, salt-tolerant *Rhizobium* strain, and compost as one such promising tool to sustainably increase alfalfa productivity under high salinity. This strategy was accomplished by assessing alfalfa growth and fitness, physiology, mineral nutrition, and an antioxidant system under normal and high-salt conditions to understand the microbiome role in plant tolerance. The effect of these biofertilizers and salinity treatment on glomalin production in soil was also evaluated. Furthermore, we suggest a novel approach to help the understanding of plant rhizosphere “microbiotics” mechanisms using the stress-induced microbiome changes by comparing salt-stressed and non-stressed plants.

## 2. Materials and Methods

### 2.1. Plant and Biofertilizer Materials

This study was carried out on alfalfa *(Medicago sativa* L. cv. Siriver). Siriver, a moderately salt-tolerant cultivar.

The native AMF (Aoufous mycorrhizal consortium) were isolated earlier from Tafilalet region located at 500 Km southeast of Marrakesh and containing a mixture of indigenous species: (i) *Glomus* sp. (15 spores/g soil), (ii) *Sclerocystis* sp. (9 spores/g soil), and (iii) *Acaulospora* sp. (one spore/g soil). The AMF consortium was multiplied by trap culture in pots using *Zea mays* L. as the host plant under controlled greenhouse conditions for three months, as described by Meddich et al. [22].

The consortium was subjected to the most probable number (MPN) test to determine its potential infectivity (1056 infective propagules per 100 g soil) [23]. Alfalfa plants were inoculated with 5 g (approximately 11 spores/g) of *Z. mays* mycorrhizal roots (AMF infection intensity = 79%) containing hyphae, vesicles, and spores to the alfalfa root system. The non-inoculated (Ct) treatment received an equal quantity of both non-inoculated (and non-mycorrhizal) *Z. mays* roots to match “organic matter” in the pots and filtered inoculum in an attempt to restore other soil free-living microorganisms accompanying the AMF. The filtrate for each pot was obtained by passing the mycorrhizal inoculum in 20 mL of distilled water through a layer of 15- to 20-μm filter papers (Whatman, GE Healthcare, Buckinghamshire, UK).

The rhizobium strain of *Ensifer meliloti RhOL1* was supplied by the Microbial Biotechnologies, Agrosciences, and Environment Laboratory (BioMAgE), Faculty of Science Semlalia, Cadi Ayyad University, Marrakesh, Morocco. *RhOL1* was isolated from *M. sativa* nodules grown in saline soils of the southeastern region of Morocco [24], showing a tolerance at 0.7 M NaCl. This rhizobial strain was tested for different plant growth-promoting rhizobacteria (PGPR) activities and biochemical characteristics such as tricalcium phosphate and potassium solubilization [25], exopolysaccharides [26], indole acetic acid [27], and nitrogen fixation (N_2_) [28]. *RhOL1* was grown on yeast mannitol (YEM) liquid medium and placed in a shaking incubator (Lab-Line Orbit Environ-shaker, Melrose Park, IL, USA) at 28 °C for 48 h. Viability counts were performed, and the inoculum was adjusted to 10^9^ cells mL ^− 1^ with an optical density of 1. The characteristics of *RhOL1* are reported in Table 1.

The compost used in this work was produced from grass waste, as described previously by Meddich et al. [29]. The compost was applied at the rate of 5% (*w*/*w* with respect to culture soil), which is the lowest concentration ensuring AMF functioning. The physicochemical and microbiological characteristics of the compost based on dry matter are as follows: pH (7.86), organic matter (527.2 g kg^−1^), organic C (306.5 g kg^−1^), total N (21.9 g kg^−1^), C/N ratio (14.0), ashes (490 g kg^−1^), NH_4_^+^ (0.03 × 10^−3^ mg g^−1^), NO_3_^−^ (0.07 × 10^−3^ mg g^−1^), NH_4_^+^/ NO_3_^−^ ratio (0.44), available P (0.25 mg g^−1^), bacterial population (1.65 × 10^8^ CFU g^−1^), fungal population (4.3 × 10^5^ colony-forming units g^−1^), and total and fecal coliforms were not detected.

### 2.2. Plant Growth Conditions

The experiment was carried out under greenhouse conditions at the Faculty of Science Semlalia, Cadi Ayyad University, Marrakesh, Morocco, with a 16/8 h day/night cycle and an average temperature of 25.5 °C, relative humidity average of 68.5%, and light of 410 μm^−2^ s^−1^.

Seeds of alfalfa *(M. sativa* L. cv. Siriver) were surface-sterilized in 95% ethanol for 30 s, and in 5% (*w*/*v*) NaClO for 10 min, and then washed five times with sterile water. Seeds sown on filter paper discs moistened in Petri dishes were incubated at 28 °C for 2 days for germination. The uniform-looking seedlings were transplanted to 2 kg plastic bags (five per bag equivalent to 15 kg/ha of alfalfa field-seeding rate) filled with sand and agricultural soil at a ratio of 1:2 (*w*/*w*). The culture soil has been previously sterilized for 3 h at 180 °C during three consecutive days. The agricultural soil has the following characteristics: pH: 8.1, electrical conductivity (EC): 73.5 μs/cm, organic matter: 0.86%, limestone: 5.58%, and available phosphorus: 7.96 mg kg^−1^. The plastic bags were placed in individual trays in the controlled greenhouse with a 16 h light (410 μmol photons s^−1^ m^−2^)/8 h dark photoperiod (25 °C during the light period and 21 °C during the dark period) and relative humidity of 40–60%.

### 2.3. Experimental Design and Treatments

The experimental design consisted of 16 treatments: un-inoculated and un-amended control plants (Ct), seedlings treated with AMF (M), or rhizobium strain (R), or compost (C), and their combinations MR, CM, CR, and CMR and grown under 0 mM and 120 mM NaCl. The experiment used eight replicates, five seedlings each, in a fully randomized design. All plants were placed randomly in the greenhouse.

Salt stress treatments consisted of 0 and 120 mM NaCl, which were applied on 3-week-old plants. To avoid the osmotic shock, NaCl concentrations were gradually increased during one transition week starting on day 1 with 30 mM NaCl for all NaCl-treated pots. Then, NaCl concentration was gradually increased by 30 mM every two days until the final concentration was reached. Excess water due to drainage was collected and added to the soil of the corresponding bags. Each bag was daily monitored and watered as necessary with distilled water or NaCl solution to keep the desired levels of salinity. After 6 weeks of salt treatment (9-week-old plants), agro-physiological and biochemical parameters were measured.

### 2.4. Plant Growth Parameters, Mycorrhizal Assessment, and Nodule Biomass

Plant height, roots length, leaf number, shoot (SDW) and root (RDW) dry weights (dried at 75 °C until the weight remained constant) were recorded at the final harvest. Nodules of alfalfa were assessed by measuring total nodule dry weight (NDW) per root.

Fresh roots were cut into 1-cm fragments, washed, and cleaned in 10% KOH at 90 °C for 30 min. The segments were acidified with 5% lactic acid for 20 min, stained with 0.05% (*w*/*v*) Trypan blue for 30 min at 90 °C according to Phillips and Hayman [30], and then microscopically assessed for mycorrhizal root colonization. The frequency of fungal structures in the root system (Fa%) and the intensity of the mycorrhizal colonization (Ma%) were evaluated in 30 randomly chosen root fragments (1-cm length) per glass slide repeated five times for each sample. Mycorrhizal parameters (Fa% and Ma%) were calculated according to McGonigle et al. [31] counted from 150 root fragments as follows:

Fa (%) = 100 × (infected root segments/total root segments)

Ma (%) = ((95 × n5) + (70 × n4) + (30 × n3) + (5 × n2) + n1)/total root segments,

where n is the number of fragments assigned with the index 0, 1, 2, 3, 4, or 5, with the following infection rates: 100 > n5 > 90; 90 > n4 > 50; 50 > n3 > 10; 10 > n2 > 1; 1 > n1 > 0.

### 2.5. Leaf Water Content

The leaf water content (LWC) of control and salt-treated leaves was determined as:

LWC (%) = 100 x (FW – DW) / FW.

FW and DW denote fresh and dry weights, respectively. Results were expressed as percentages.

### 2.6. Determination of Nutrient Concentrations in Plants

The P content in leaves (mg g^−1^ DW) was quantified using the Olsen method [32]. The Na^+^, K^+^, and Ca^2+^ content were measured by flame spectrophotometer (JENWAY, PFP7) as described by Wolf [33]. The nitrogen (N) content was determined by colorimetry after the Kjeldahl digestion, and chlorine (Cl^−^) content was measured using the silver nitrate (AgNO_3_) titration method [34].

### 2.7. Photosynthesis-Related Performance

The efficiency of photosystem II was evaluated by measuring chlorophyll fluorescence in the third youngest, fully expanded, and attached leaves using a portable fluorometer (Opti-sciences OSI 30p) for dark-adapted leaves. Before measurements, leaves were acclimated to dark for 30 min using leaf clips. Chlorophyll fluorescence variables were recorded: Initial (F_0_), maximum (F_m_), variable (F_v_ = F_m_ − F_0_) fluorescence as well as F_v_/F_m_ ratio. Four plants (four records from different parts of each leaf) for each treatment were used for measurements.

Stomatal conductance (g_s_) was measured using a porometer system (Leaf Porometer LP1989, Decagon Device, Inc., Washington, DC, USA) in the second youngest leaf on four replications per treatment on a sunny day.

The concentration of total chlorophyll was determined according to the method described by Arnon [35]. The photosynthetic pigment was extracted from the fresh tissue (100 mg) powder using cold acetone 80%. Following centrifugation at 10,000× *g* for 10 min, supernatant absorbance was read at 480, 645, and 663 nm using a UV–visible spectrophotometer (UV-3100PC spectrophotometer, VWR).

### 2.8. Determination of Electrolyte Leakage, Malondialdehyde, and Hydrogen Peroxide Content

Electrolyte leakage (EL) was determined according to the method described by Lutts et al. [36]. Briefly, alfalfa samples were washed three times with deionized water to remove any surface-adhered electrolytes and cut into 2-cm pieces. Samples were placed in closed vials containing 10 mL of deionized water and incubated on a shaker at 30 °C for 6 h. After incubation, electrical conductivity (EC) of the solution (EC_1_) was determined using a conductivity meter (Hannah Instruments HI8820 N). Then, the samples were incubated for 20 min at 120 °C, and the EC was measured again after equilibration at 25 °C as EC_2_. EL was calculated as: EL (%) = (EC_1_/EC_2_) × 100.

Malondialdehyde (MDA) was determined according to the method described by Madhava Rao and Sresty [37]. In brief, lipid peroxides were extracted from the frozen leaf (0.25 g) powder subsamples with 10 mL of 0.1% trichloroacetic acid (TCA). After centrifugation (18,000× *g* for 20 min), the chromogen was formed by mixing 1 mL of supernatant with 2 mL of 20% TCA containing 0.5% thiobarbituric acid (TBA). The mixture was incubated at 100 °C for 30 min, and the reaction was stopped by placing the tubes in an ice bath. The chromogen formed was measured at 450, 532, and 600 nm, and the MDA content was calculated as follows: [MDA] = 6.45 (A_532_ − A_600_) − 0.56A_450_.

Hydrogen peroxide (H_2_O_2_) concentration was determined spectrophotometrically, according to Velikova et al. [38]. Frozen leaf (0.25 g) powder was homogenized with 5 mL 10% (*w*/*v*) TCA and then centrifuged at 15,000× *g* for 15 min at 4 °C. The supernatant (0.5 mL) was recovered to determine the content of H_2_O_2_ and 0.5 mL of potassium phosphate buffer (10 mM, pH 7) and 1 mL of iodic potassium (1 M) were added. After 1 h of incubation, the absorbance was read at 390 nm and plotted against a standard H_2_O_2_ curve. The blank was made by replacing the sample extract by 10% TCA.

### 2.9. Determination of Proline and Antioxidant Enzymes Concentrations in Plants

Free proline content was determined according to the method described by Carillo et al. [39] Briefly, 100 mg of leaf plant material was homogenized in 4 mL of 40% ethanol, and the homogenate was kept overnight at 4 °C. The solution (0.5 mL) was reacted with 1 mL of a mixture containing 60% acetic acid, 1% ninhydrin, and 20% ethanol in a test tube for 20 min at 90 °C, and then the reaction was stopped by submerging the tubes in an ice bath. The absorbance was read at 520 nm.

Frozen leaf powder subsamples (0.1 g) were homogenized in a cold mortar with 4 mL of 1 M phosphate buffer (pH 7) containing 5% polyvinylpolypyrrolidone (PVPP). The homogenate was centrifuged at 18,000× *g* for 15 min at 4 °C, and the supernatant was used to measure the antioxidant enzyme activity [40]. Total soluble proteins were determined according to the technique described by Bradford [41].

Superoxide dismutase (SOD, EC 1.15.1.1) activity was assayed by Beyer and Fridovich method [42]. One unit of SOD activity was defined as the amount of enzyme leading to 50% inhibition of nitro blue tetrazolium (NBT) reduction at 25 °C. The activity of SOD was expressed at unit min^−1^ mg protein ^−1^.

Catalase (CAT, EC 1.11.1.6) activity was measured by monitoring the consumption of H_2_O_2_ substrate at 240 nm for 3 min [43]. The reaction mixture consisted of 100 μL of enzyme extract, potassium phosphate buffer (0.1 M, pH 7.0), 0.1 mM EDTA, and 20 mM H_2_O_2_ in a 2 mL volume.

Ascorbate peroxidase (APX, EC 1.11.1.11) activity was assayed as a decrease in absorbance at 290 nm for 1 min according to Amako et al. method [44]. The assay solution contained 100 μL of extract sample, 50 mM potassium phosphate buffer (pH 7.0), 0.5 mM H_2_O_2_, and 0.1 mM ascorbate. The reaction was initiated by adding the enzyme extract and the decrease in absorbance was recorded.

### 2.10. Soil Analysis

At the harvest, four soil samples of each treatment were taken near the roots, air-dried at room temperature, and sieved at 2.0 mm for the subsequent analyses. The electrical conductivity (EC) was measured in a 1:2 (w:v) aqueous solution using a conductivity meter HI-9033 (Hanna Instruments, Padova, Italy). Total glomalin-related soil protein (T-GRSP) and easily extractable GRSP (EE-GRSP) were examined according to Cornejo et al. [45]. T-GRSP was extracted from 2 g of soil with 8 mL 50 mM sodium citrate (pH 8.0), followed by autoclaving for 1 h at 121 °C. For EE-GRSP, samples of 2 g soil were extracted with 8 mL of 20 mM sodium citrate (pH 7.0), followed by autoclaving for 30 min at 121 °C. For both fractions, the supernatants were separated by centrifugation at 10,000× *g* for 1 h. The T-GRSP extraction was carried out five times until the solution was straw-colored. Protein content in the crude extracts was determined according to the Bradford method [41].

### 2.11. Statistical Analysis

Data are presented as mean ± SE (standard error) of at least four independent biological replicates per treatment. Statistical analysis was carried out with the software package SPSS 23.0 for Windows. All results were subjected to a multivariate analysis of variance (MANOVA) for the main factors (Salinity × AMF × *Rhizobium* × Compost) and their interactions (see Table S1). Different lower cases indicate significant differences among treatments using the post hoc Tukey’s honestly significant difference test at *p* < 0.05. Growth, agro-physiological, biochemical data, and their association with treatments were subjected to principal component analyses (PCA) using XLStat software v. 2019. Loading values, percentage contributions of principal component (PC), and percentage contributions of the variables to the PC are shown in Table S2.

## 3. Results

### 3.1. Effect of Salinity and Biofertilizers on Symbiotic Development, Growth Parameters, and Shoot Water Status

Non-inoculated plants did not exhibit AMF root colonization nor nodulation on their root systems (Table 2). The plants inoculated with native AMF Aoufous without compost, and rhizobium showed the higher root colonization intensity compared to plants treated with compost and/or rhizobium under both normal and stressed conditions. The application of the rhizobium strain (MR treatment) showed no effect on root mycorrhizal frequency (Fa = 85%) compared to the single M inoculation, while it increased ca. 48% the infection intensity under normal condition. The addition of the compost amendment decreased the mycorrhizal parameters (Fa% and Ma%) more than the un-amended R and MR treatments, while the nodule weight increased in CR and CMR. In the presence of compost, the plants inoculated with native AMF or rhizobium showed the higher root colonization intensity compared to plants treated with compost or rhizobium. Under salt stress, the frequency and intensity of mycorrhization decreased in all the treatments independently of the presence or absence of compost. Interaction between salt stress, compost, AMF, and the rhizobial strain was significant for these two parameters (*p* < 0.05) (Table S1).

**Figure 1:**
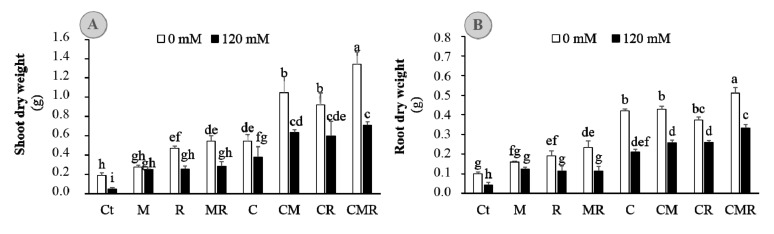
(**A**) Shoot and (**B**) root dry weights of alfalfa plants grown without (0 mM NaCl; white bars) or with (120 mM NaCl; black bars) salt stress and submitted to different biofertilizers treatments; Ct: untreated control, M: inoculated with arbuscular mycorrhizal fungi, R: inoculated with rhizobium strain, C: amended with compost, MR: inoculated with the mixture AMF+rhizobium, CM: amended with compost and inoculated with the AMF, CR: amended with compost and inoculated with rhizobium, and CMR: amended with compost and inoculated with the mixture AMF+ rhizobium. Means (±SE) within the same graph, followed by different letters are significantly different at *p* < 0.05.

Previously, the compost-derived and rhizosphere-enriched isolates were associated with plant growth promotion. Therefore, the compost and/or native AMF and/or salt-tolerant rhizobium strains were tested for their ability to promote alfalfa growth under high salinity. Alfalfa plants were exposed to single, dual, or tri-combination, and plant height, roots length, leaf number, and leaf water content were determined after co-cultivation (Table 2). All the soil amendments increased these four parameters under both unstressed and stressed conditions, with the tripartite combination CMR being the most effective compared to untreated and un-inoculated control plants (Table 2 and Table S1). The growth of control plants was inhibited under saline condition. The addition of symbionts and/or compost overcame the slower plant growth. Indeed, Alfalfa plants inoculated with the tripartite combination had ca. 60, 71, and 81% higher plant height, roots length, and leaf number, respectively, compared to the control plants under both NaCl conditions. Total biomass yield was significantly affected by salt stress (Figure 1), 120 mM NaCl reduced substantially plant biomass either on the shoot or dry root matters. When salt stress was applied, biomass was reduced by 75% and by 58% on a DW basis in shoot and root, respectively, as compared to unstressed plants (0 mM NaCl, Figure 1) (*p* < 0.001; Table S1). However, under normal and stressed conditions, the application of single, bi-, and tripartite combinations of organic compost and biologic (AMF and/or rhizobium RhOL1) amendments showed positive effects by promoting alfalfa biomass to a greater extent than in un-inoculated and un-amended plants (Figure 1). Under normal condition, AMF did not significantly affect root or shoot biomass per se, but the shoot/root ratio was higher for the inoculated plants, this ratio being significantly lower in control treatment after salt stress imposition (data not shown). These results indicated that biofertilizers application could promote the growth of alfalfa plants exposed to salinity.

### 3.2. Effect of Salinity and Biofertilizers on Shoot Ion Composition

**Figure 2:**
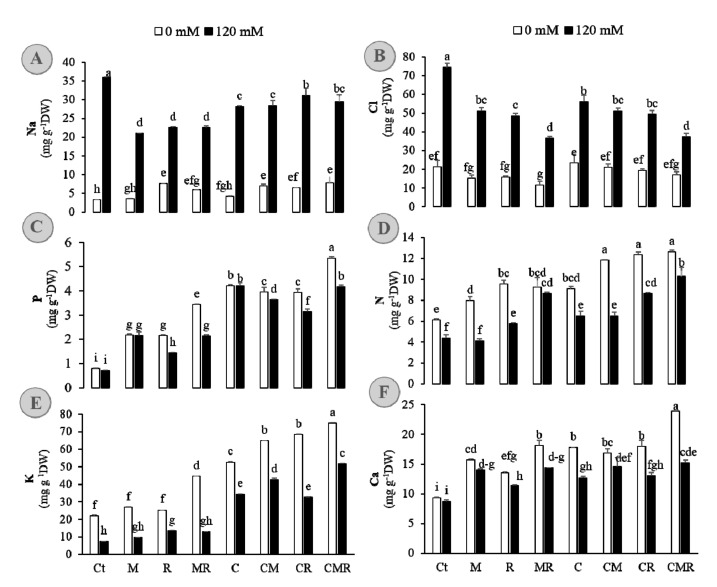
(**A**) Sodium, (**B**) chloride, (**C**) phosphorous, (**D**) nitrogen, (**E**) potassium, and (**F**) calcium in alfalfa shoots grown without (0 mM NaCl; white bars) or with (120 mM NaCl; black bars) salt stress and submitted to different biofertilizers treatments; Ct: untreated control, M: inoculated with arbuscular mycorrhizal fungi, R: inoculated with rhizobium strain, C: amended with compost, MR: inoculated with the mixture AMF+rhizobium, CM: amended with compost and inoculated with the AMF, CR: amended with compost and inoculated with rhizobium, and CMR: amended with compost and inoculated with the mixture AMF+rhizobium. Means (± standard error) within the same graph followed by different letters are significantly different at *p* < 0.05.

We assayed the Na^+^, Cl^−^, N, P, K^+^, and Ca^2+^ contents in shoots of alfalfa treated or not with biofertilizers under salinity, since the degree of tolerance depends on their uptake and translocation. Under control condition, shoot Na^+^ and Cl^−^ were comparable and remained similarly low among treatments (Figure 2A,B), whereas under salt stress, they were significantly higher in Ct shoot than in treated and inoculated ones. Salinity stress increased Na^+^ and Cl^−^ concentrations in Ct shoots relative to the other treatments, reaching values between 1.2× to 1.4× in Na^+^ and 1.4× to 2× that in compost-treated and/or inoculated plants (Figure 2A,B). Under salinity, the lowest values of Na^+^ were found in the rhizosphere-enriched isolates (M, R, and MR) and in MR and CMR for Cl^−^, which recorded ca. 50% inhibition absorption of these ions than untreated plants. The nutrient status of alfalfa leaves (P, N, K^+^, and Ca^2+^) was significantly increased by all the applied biofertilizers, independently of the presence or absence of salt stress, compared with non-treated stressed plants (Figure 2C–F). The treatments’ types, salinity, and the interaction effects were highly significant for the concentrations of all shoot ions (Table S1). Under salinity, the highest values of N, K^+^ and Ca^2+^ were recorded in the tripartite combination CMR. It was 135% for N, 200% for K^+^, and 74% for Ca^2+^ in CMR shoots compared to the Ct level. Together with CMR, the compost-derived treatment accumulated significantly more P than the other treatment under salinity (Figure 2C), reaching 5× than untreated stressed plants.

### 3.3. Biofertilizers Improve the Photosynthesis-Related Performance in Alfalfa Grown under Normal and High-Salinity Conditions

**Figure 3:**
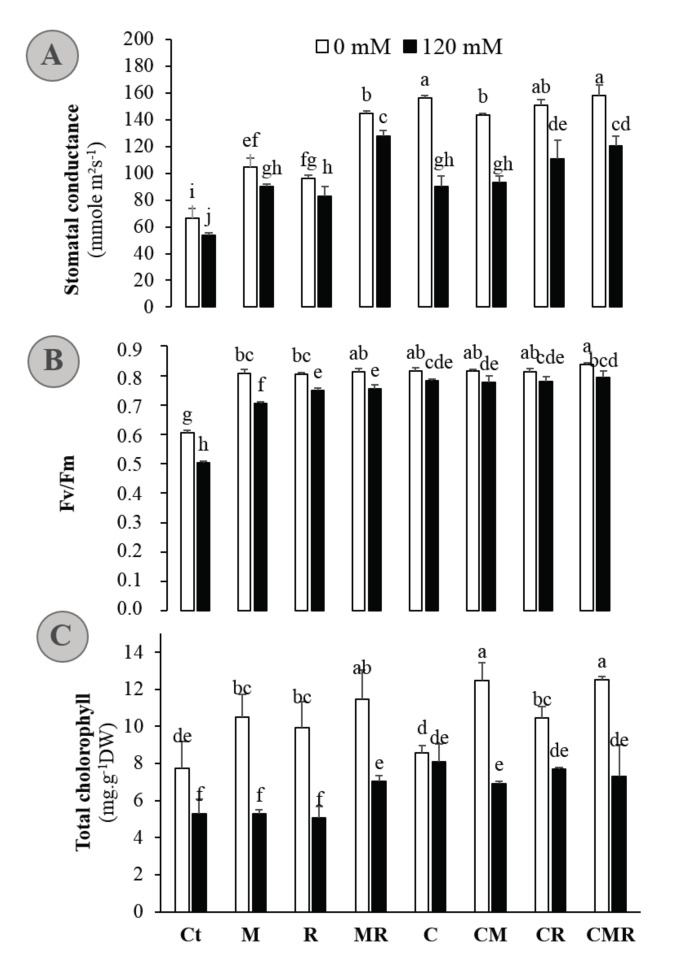
(**A**) Stomatal conductance, (**B**) chlorophyll fluorescence, and (**C**) total chlorophyll concentration of alfalfa plants grown without (0 mM NaCl: white bars) or with (120 mM NaCl: black bars) salt stress and submitted to different biofertilizers treatments; Ct: untreated control, M: inoculated with arbuscular mycorrhizal fungi, R: inoculated with rhizobium strain, C: amended with compost, MR: inoculated with the mixture AMF + rhizobium, CM: amended with compost and inoculated with the AMF, CR: amended with compost and inoculated with rhizobium, and CMR: amended with compost and inoculated with the mixture AMF + rhizobium. Means (± standard error) within the same graph followed by different letters are significantly different at *p* < 0.05.

We evaluated the protective role of compost-derived and rhizosphere-enriched isolates application on photosynthetic machinery under normal and salinity conditions. The overall results revealed that salinity caused a significant (*p* < 0.001) decline in stomatal conductance (g_s_), the quantum efficiency of photosystem II (F_v_/F_m_), and total chlorophyll (Chl) (Figure 3). Indeed, the exposure to salt stress in untreated plants declined g_s_ significantly by 19%, F_v_/F_m_ by 17%, and Chl by 32% compared with untreated and non-stressed alfalfa plants. The application of different biofertilizers significantly improved these parameters compared to non-treated plants, independently of the salinity. Plant inoculation with AMF (*p* < 0.001) and/or rhizobium (*p* < 0.001) amended or not with the compost (*p* < 0.001) yielded an improvement in stomatal conductance and efficiency of photosystems I and II in coping with salinity. Under salinity, the application of biofertilizers increased g_s_ by ca. 40 to 62%, F_v_/F_m_ by ca. 30 to 38%, and total Chl by ca. 30 to 45% as compared to untreated plants. Significant interaction among SS (salt stress) × M × R × C treatment was observed for total chlorophyll content (*p* < 0.05) (Table S1).

### 3.4. Biofertilizers Mitigate the Negative Impacts of Salinity by Decreasing the Electrolyte Leakage, Malondialdehyde, and Hydrogen Peroxide Content in Alfalfa Grown under High-Salinity

**Figure 4:**
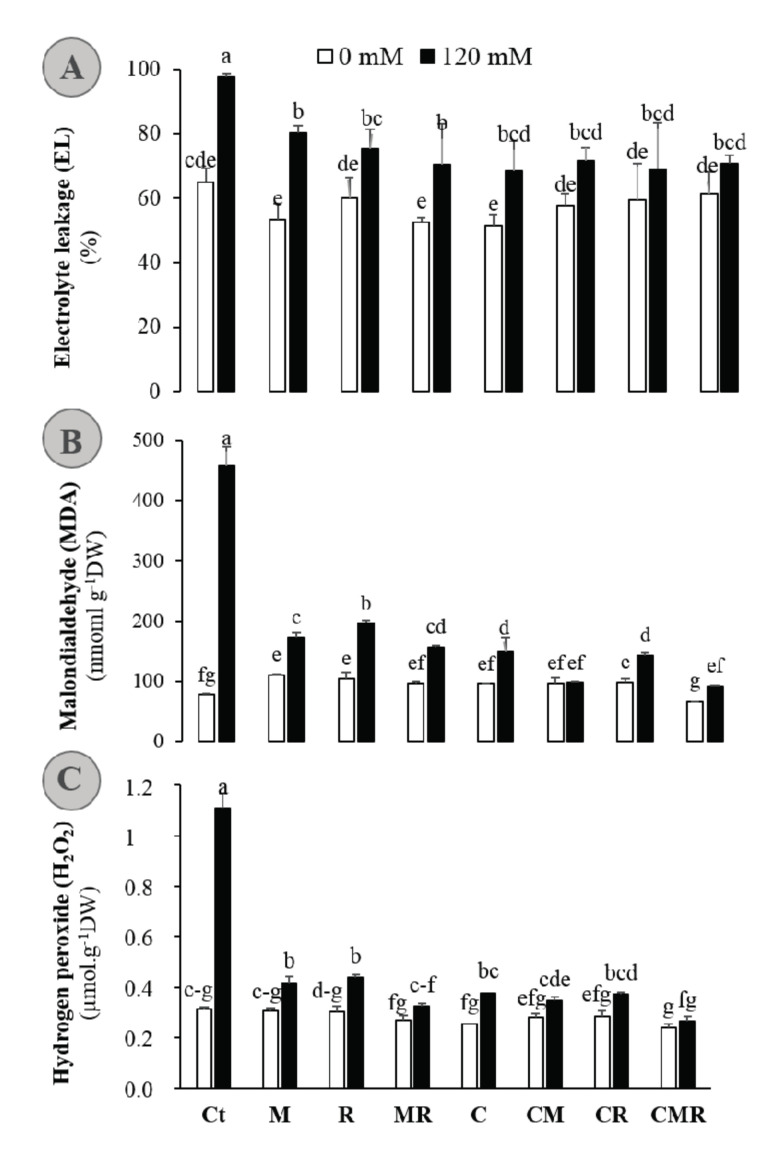
(**A**) Electrolyte leakage, (**B**) malondialdehyde (MDA), and (**C**) hydrogen peroxide (H_2_O_2_) content in the shoot of alfalfa plants grown without (0 mM NaCl: white bars) or with (120 mM NaCl: black bars) salt stress and submitted to different biofertilizers treatments; Ct: untreated control, M: inoculated with arbuscular mycorrhizal fungi, R: inoculated with rhizobium strain, C: amended with compost, MR: inoculated with the mixture AMF + rhizobium, CM: amended with compost and inoculated with the AMF, CR: amended with compost and inoculated with rhizobium, and CMR: amended with compost and inoculated with the mixture AMF + rhizobium. Means (± standard error) within the same graph followed by different letters are significantly different at *p* < 0.05.

To test if the AMF and/or bacterial strains with or without compost have the potential to help to withstand the salt stress, we evaluated their ability to modify plant physio-biochemical attributes and the oxidative defense system. As important indicators of oxidative damage under environmental stresses, electrolyte leakage (EL), malondialdehyde (MDA), and hydrogen peroxide (H_2_O_2_) were measured (Figure 4). EL, MDA, and H_2_O_2_ increased considerably (*p* < 0.001; Table S1) in the salt-stressed alfalfa plants. Under high salinity, enrichment of rhizosphere by compost and/or selected members of the microbiome was effective in lowering the EL, MDA, and H_2_O_2_ compared with untreated salt-affected alfalfa plants. While all the single, bi- and tripartite combinations of biofertilizers reduced EL in an equal manner ca. −25% compared to the stressed control, the CMR composition in alfalfa plants displayed the lowest accumulation of MDA and H_2_O_2_, −54 and −45%, respectively, compared with untreated Ct plants (*p* < 0.05).

### 3.5. Biofertilizers Enhance Osmolytes and Oxidative Defense System in Alfalfa Grown under High Salinity

**Figure 5:**
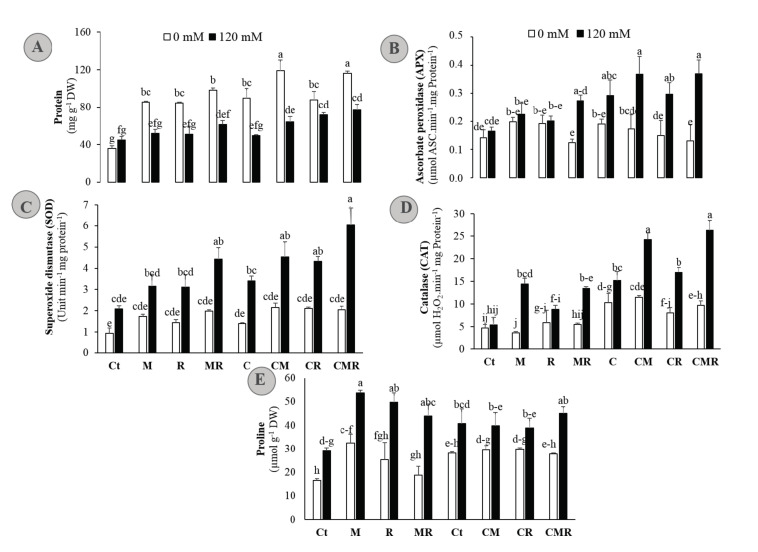
(**A**) Protein concentration, (**B**) ascorbate peroxidase (APX), (**C**) superoxide dismutase (SOD), and (**D**) catalase (CAT) activity, and (**E**) proline content in the shoot of alfalfa plants grown without (0 mM NaCl: white bars) or with (120 mM NaCl: black bars) salt stress and submitted to different biofertilizers treatments; Ct: untreated control, M: inoculated with arbuscular mycorrhizal fungi, R: inoculated with rhizobium strain, C: amended with compost, MR: inoculated with the mixture AMF + *rhizobium*, CM: amended with compost and inoculated with the AMF, CR: amended with compost and inoculated with *rhizobium*, and CMR: amended with compost and inoculated with the mixture AMF + *rhizobium*. Means (± standard error) within the same graph followed by different letters are significantly different at *p* < 0.05.

To dissect how salt stress tolerance is affected by organic and/or biologic biofertilizers, the antioxidant system in alfalfa plants was measured after treatments with 120 mM NaCl. Under 0 mM NaCl, alfalfa plants treated or not with biofertilizers showed no significant differences of the antioxidant enzymes activities (Figure 5B–D). In contrast, the presence of biofertilizers increased the protein and proline concentrations in free-NaCl condition (Figure 5A,E). Compared to the untreated plants, 120 mM NaCl did not result in significant oscillations in protein concentration when biofertilizers were applied alone (Figure 5A). Under salt stress condition, the activities of APX, SOD, and CAT were greatly enhanced by biofertilizers treatments. Noticeably, bi- and tripartite combinations effectively improved the activities of these three antioxidant enzymes compared with non-treated seedlings under salt stress (Figure 5B–D). Proline usually acts as an important osmotic regulator in plants. Compared to the control plants, the levels of proline significantly increased in the leaves under salt stress. Application of biofertilizers further increased the contents of this osmotic regulator under salt stress (Figure 5E). The significant increase in enzymatic and non-enzymatic antioxidants level following the application of biofertilizers under NaCl treatment suggested the role of biofertilizers in alfalfa responses to salt stress and their involvement in alleviating salt stress-induced oxidative damage.

### 3.6. Soil Analysis

**Figure 6:**
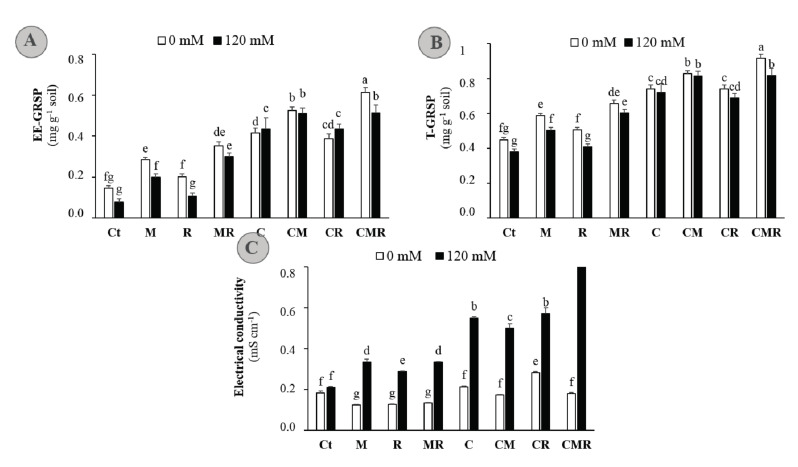
(**A**) Easily extractable glomalin-related soil protein (EE-GRSP), (**B**) total glomalin-related soil protein (T-GRSP), and (**C**) electrical conductivity in soil at harvest time of alfalfa plants grown without (0 mM NaCl: white bars) or with (120 mM NaCl: black bars) salt stress and submitted to different biofertilizers treatments; Ct: untreated control, +M: inoculated with arbuscular mycorrhizal fungi, +R: inoculated with rhizobium strain, C: amended with compost, M+R: inoculated with the mixture AMF + rhizobium, C + M: amended with compost and inoculated with the AMF, C+R: amended with compost and inoculated with rhizobium, and C + M + R: amended with compost and inoculated with the mixture AMF + rhizobium. Means (± standard error) within the same graph followed by different letters are significantly different at *p* < 0.05.

To explore the impact of biofertilizers in alfalfa growing soil under salt stress, soil parameters were examined. The results revealed that salt stress negatively affected EE-GRSP (*p* < 0.01) and T-GRSP (*p* < 0.001) in untreated plants Ct, M and R applied alone, or in combination (MR) (Figure 6A,B). The compost treated plants were generally equivalent to their control without stress. While salt-tolerant rhizobium strain displayed no apparent effects on EE-GRSP and T-GRSP under normal and high-salinity conditions, the AMF, compost, and all the combinations improved these two parameters as compared with non-inoculated and non-amended plants, under both normal and salt stress conditions. Consistently, under salt stress condition, the highest value of GRSP was noted in CM and CMR treatments with an enhancement of 249 and 250%, respectively, as compared to the control plants. When salt treatment was applied, biofertilizer-treated plants presented significantly higher electrical conductivity in their soils (*p* < 0.001), being the highest value (+280% than Ct) recorded in CMR treatment (*p* < 0.001). These data indicated that biofertilizers might have essential effects on soil nutrients availability under stressful conditions by the EC and ability of glomalin in sequestering soil nutrients, thereby increasing their availability according to the soil conditions.

### 3.7. Principal Component Analysis

**Figure 7:**
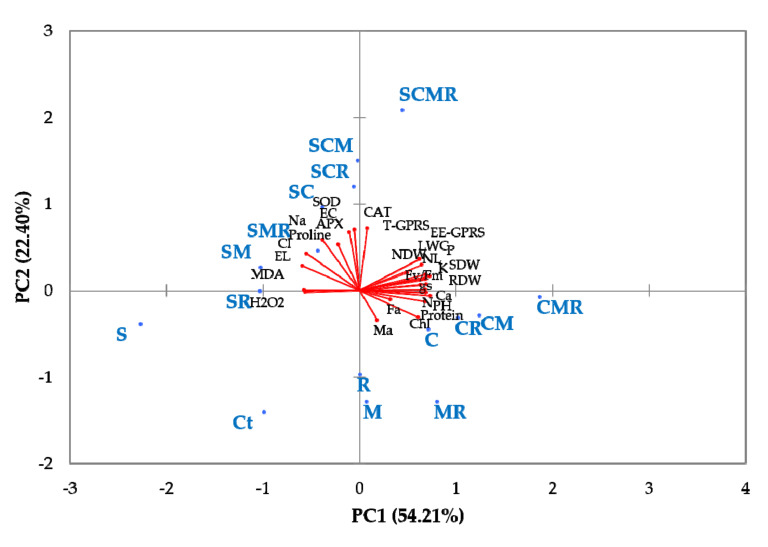
Principal component analysis (PCA) of alfalfa grown without (0 mM NaCl) or with (120 mM NaCl) salt stress and submitted to different biofertilizers treatments. Ct: untreated control, M: inoculated with arbuscular mycorrhizal fungi, R: inoculated with rhizobium strain, C: amended with compost, MR: inoculated with the mixture AMF + rhizobium, CM: amended with compost and inoculated with the AMF, CR: amended with compost and inoculated with rhizobium, and CMR: amended with compost and inoculated with the mixture AMF+rhizobium. SDW: shoot dry weight, RDW: root dry weight, PH: plant height, RL: roots length, NL: leaf number, Fa: AMF infection frequency, Ma: AMF infection intensity, NDW: nodule dry weight, LWC: leaf water content, P: phosphorus uptake, N: nitrogen uptake, Na: sodium uptake, Cl: chlore uptake, K: potassium uptake, Ca: calcium uptake, F_v_/F_m_: chlorophyll fluorescence, g_s_: stomatal conductance, Chl: total chlorophyll content, EL: electrolyte leakage, MDA: malondialdehyde content, H_2_O_2_: hydrogen peroxide content, Proline: proline content, Protein: protein content, SOD: superoxide dismutase activity, CAT: catalase activity, APX: ascorbate peroxidase activity, EE-GPRS: easily extractable glomalin-related soil protein, T-GPRS: total extractable glomalin-related soil protein, EC: electrical conductivity.

To understand the relationship between the biofertilizers, salinity, and predictor parameters, a PCA analysis was carried out to establish the variables with a prevalent influence on plant growth and stress tolerance (Figure 7 and Table S2). Data showed that PC1 explained 55.3% and PC2 explained 21.7% of the total variance. Single or combined biofertilizers treatments were separated from the control and salt stress. Treatments resulting in more growth and biomass production are on the right side of the first axis (PC1). Compost alone or combined with microbial inoculants treatments were positively linked with growth, AMF infection, NDW, LWC, photosynthetic traits, nutrient concentration (N, P, K, and Ca), protein, and glomalin content in the soil.

In contrast, the untreated controls showed lower growth, since the salt stress control was negatively affected (left lower panel of the first axis). In parallel, the treatments on the vertical axis corresponded to intermediate growth, as well as proline, chlorophyll, and antioxidant enzymes production. Consistently, compost- and inoculated-treated plants were nearly equivalent to those parameters. In addition, the analysis showed that the triple combination CMR under salinity, observed in the upper right panel, was significantly separated from the other stresses and treated plants, being positively correlated with PC1 and PC2 components. The overall results of this analysis showed that biofertilizers were the best treatments in terms of plant growth under unstressful conditions, and they were closely related to the antioxidant defense system under high salinity, which could promote more comprehensive understanding in the protective effects of biofertilizers in alfalfa under salt stress.

## 4. Discussion

Salinity is one of the significant causes limiting the global production of food and forage. Therefore, it is essential to find strategies allowing plants to withstand salt stress, focusing on biomass, to ensure food security and mitigate damaging the grazing areas of livestock. To our knowledge, this is the first study to summarize the extent to which the effect of single and multiple combinations of symbiosis with native AMF and/or halotolerant *per se* rhizobia *RhOL1*, and/or compost-based amendments are crucial to improving *M. sativa* fitness and health under high salinity. Therefore, this strategy could be used for designing the functionally reliable system as “plant probiotics”. Our finding revealed that the alfalfa inoculated with the soil-derived bacteria and fungi under salinity had a significantly higher above- and below-ground biomass, water status, and mineral nutrients than untreated plants, which suggest the positive protective role of these amendments against salt stress.

The results showed that these microorganisms were more effective in compost-amended soil, under both normal and salt stress conditions, despite the reduction of AMF root colonization. The data confirmed that the stimulation of plant growth is not always related to the degree of AMF root colonization, as previously reported by Cavagnaro et al. [46]. Earlier studies showed that the dual application of compost and AMF could have positive [13] or negative [3] effects on the levels of AMF root colonization. However, the mechanisms that underpin these effects remain to be elucidated [46]. The formation of AMF is strongly affected by edaphic conditions and soil management practices. The supply of organic forms of nutrients, such as compost, generally results in a decrease in AMF colonization [3]. Despite the reduction of AMF colonization in the presence of compost, Cavagnaro et al. [46] reported that it is essential to take into consideration the root length colonized, since a decrease in percent colonization of roots by AMF can be accompanied by a massive increase in root biomass with compost addition. We hereby confirm that our root biomass data support the assumption. Thus, the positive effects of compost on the formation of AMF symbiosis may be masked by the decrease in percent colonization [46]. In contrast, *RhOL1* strain increased the AMF root colonization regardless of the salinity conditions. This beneficial effect suggests a possible activity of the rhizobial strain as “MHB” (Mycorrhization Helper Bacteria), which can stimulate the mycorrhization of plants by different mechanisms such as increasing AMF–root contact [47]. In addition, in legumes, *Rhizobium* Nod factors and mycorrhizal Myc factors are suggested to be perceived by host roots for the activation of signal transduction or common symbiosis (SYM) pathways [48], which prepares the host plant to bring about changes at the molecular and anatomical level with the first contact of fungal hyphae. Amendment with compost and AMF inoculation increased the nitrogen fixation attributes, including the weight of nodules allowing additional uptake and assimilation of nitrogen in roots when compared to salt-stressed plants (Table 2 and Figure 2). The latter is in the vein of previous studies showing the positive effects of organic amendment and AMF on nodule growth, nitrogenase activity, and leghemoglobin content leading to a greater N uptake in several crops [49]. The increase of nodule dry weight is due to the favorable effect of both AMF and compost on improving plants nutrient status such as P, which in turn allows the host plant to form the nodules and fosters N fixation by the rhizobia bacteria [50]. Liu et al. [50] showed that AMF-increasing P availability to plants improves the quantity and quality of the nodulation.

In the absence of biofertilizers, the alfalfa biomass production is adversely affected by increasing NaCl stress level (Table 2). This reduction in untreated plants is owing to both the osmotic phase during which growth inhibition is mainly due to the difficulty for the plant to absorb soil water, low LWC (Table 2), and an ionic phase due to the toxic effect of the salt within the plant, which is observed with the higher Na^+^ and Cl^−^ (Figure 2). Interestingly, we found that the tripartite combination CMR significantly promoted alfalfa growth under both control and saline conditions as compared to the absolute control; plants without amendments and inoculation (Table 2). Physiologically, the plants treated with CMR maintain their fully hydrated state under saline condition, which could at least partially have rapid and extensive effects on cell expansion, cell division, stomatal opening, and maintain regular rates of transpiration. It seems that alfalfa associated with AMF and *RhOL1* in compost-treated soil possesses an advantage against NaCl stress because more biomass was produced above-ground and also allocated to the roots under the high salinity. This observation could lead to an increase in root surface for water extraction without penalizing the aerial part. Campanelli et al. [8] showed the importance of the AMF-colonizing root system in providing a better water supply to the plant likely by increasing root density, thereby alleviating salt stress in alfalfa. Other studies showed that the association with AMF amends the plants’ water regulation by increasing hydraulic conductivity via root aquaporin (AQP) genes up-regulation, stimulating osmolytes or triggering hormonal signalings such as abscisic acid (ABA)-mediating stomatal conductance, jasmonic acid, and strigolactones [51]. In addition, we observed a massive increase in shoot water content in the presence of the symbiotic association with microbes and compost. The capacity of plants to maintain turgor under high salinity is a feature linked with NaCl tolerance, as it helps maintain cell growth and stomatal opening to allow photosynthesis. Our observation of the ability of biofertilizers-treated alfalfa to maintain turgor under salt stress could be directly linked to the mitigation of salt toxicity by a dilution effect. Salinity improved the Na^+^ and Cl^−^ ion content in alfalfa plants. However, while Na^+^ and Cl^−^ concentration are crucial for plant growth, an excess of their concentrations in cytoplasm seems to be particularly damaging to the plant metabolism [3,10]. Reducing the accumulation of Na^+^ and Cl^−^ ions in tissues is a mechanism adopted by plants to combat saline stress. In this study, the lowest values of these minerals were obtained when the biofertilizers were applied (Figure 2). A decrease in the contents of Na^+^ and Cl^−^ ions may be due to a change in the selection of ions by membranes, and thus, decreased levels of Na^+^ may cause increased K^+^ transport into the cell [52]. Our observations demonstrated that this phenomenon exists in alfalfa under salt stress following biofertilizers application. The Na^+^ content and Na^+^/K^+^ ratio in alfalfa were divergent between biofertilizers-treated and biofertilizers-free plants and therefore may represent the effects of “basic” strategies related to salt tolerance or susceptibility. Rana et al. [53] showed that under salt stress, the susceptible plants had more Na^+^ densely localized in shoot tissue. Our results suggest that the accumulation of more K^+^ with fewer Na^+^ in inoculated plants with compost would be mediated by a mechanism of K^+^ influx and Na^+^ efflux. Salinity is known to decrease the macro- and micronutrients concentrations in leaf tissue, and their accumulation is commonly used to evaluate the ability of the plant to tolerate the salt [3]. Under salt stress, the content of N was reduced in alfalfa, and the untreated plants were the most affected. Inoculation with rhizobium and compost enhanced the concentration of this ion in alfalfa plants under salinity stress.

In our investigation, Na^+^ and Cl^−^ concentrations increased in leaf, while N, K^+^, and Ca^2+^ concentrations decreased under salinity (Figure 2 and Table S1). Increased salinity lead to a loss of K^+^ due to the depolarization of membranes and loss of Ca^2+^ due to the displacement by Na^+^ ions [1]. Ekinci et al. [54] showed that the increase in these mineral nutrients is due to the modification in the strength and structure of cellular membranes and the optimal environment provided by the compost for the growth of rhizobia and AMF, which in turn contribute to nutrient bioavailability through N fixation and the solubilization of inorganic forms of minerals such as P and K [17]. Applied biofertilizers enhanced the P and Ca^2+^ ions content in alfalfa. Salvioli et al. [55] showed that *Glomus versiforme* possesses inorganic phosphate transporters on its hyphae involved in the direct absorption of P, and a glutamine synthase gene was found in *Rhizophagus intraradices*, which strengthens the possibility of N metabolism at the hyphae level that can be transported later to the plant. Indeed, an increase in P content by AMF could increase nitrogenase enzyme activity, leading to a higher N_2_ fixation of rhizobial symbiont and in return a better mycorrhizal development [56]. P content in the alfalfa plants co-inoculated with rhizobium and native AMF was much higher compared with single inoculated plants, which indicates that AMF colonization stimulated nodule formation and functioning. So far, Ca^2+^ is supposed to be the hub of secondary messengers via Ca^2+^ spiking in the nuclear region of root hairs [57]. Compost is rich in several mineral elements including N, P, K^+^, and Ca^2+^, and it maintains the soil pH [7]. Compost amendment increased the biomass and yield by increasing the uptake of essential mineral elements in crops [58]. Our data showed that the use of compost supplemented with AMF and *RhOL1* was useful for N, P, K^+^, and Ca^2+^ uptake either under stress or normal conditions, albeit this treatment decreased the AMF colonization. *RhOL1* is more adapted to salinity and could likely help maintain a higher N_2_ fixing capacity under NaCl stress. Previous studies on legumes reported that stress-tolerant rhizobium strains maintained a higher level of N fixation as well as higher biomass yield [9]. The induction of better root growth in this treatment may facilitate the mineral uptake by plants. In addition, the *RhOL1* strain has the ability to produce indole-3-acetic acid (IAA), which might induce the modulation of the root architecture [59]. At the same time, AMF and compost have been shown to contribute to the stability of soil aggregates resulting in soil physicochemical and biological properties improvement, and hence a better root growth and nutrition [13,60]. Reports discussing the combined effect of AMF, rhizobia, and/or compost are very scanty, and it is worth noting that tripartite combination treatment mitigated the negative salinity impact more than their individual treatment.

Beneficial root-colonizing microorganisms and organic amendment can affect the soil traits, which indirectly influence plants’ growth and yield. A critical factor in the contribution of AMF to soil aggregation is the production of glomalin (GRSP), which is a glycoprotein substance enhancing soil structure by holding soil particles into aggregates and stabilizing them, and it provides benefits through improving soil aeration and drainage as well as generating higher microbial activity [16]. Glomalin is also useful in sequestering various toxic elements, including Na^+^ ions [60]. Moreover, the exopolysaccharides (EPS) produced by the *RhOL1* strain (Table 1) could also play an essential role in the soil structure and mineral nutrition uptake [61]. Indeed, it has been shown that EPS may help plants cope with salt stress by both maintaining water film and binding Na^+^ ions and thus to hold them from absorption [61]. In this study, root-derived microbes and/or compost amendments, especially CMR, increased the soil EC as compared to untreated plants under salt stress (Figure 6). Thus, this treatment might accumulate salt in rhizospheric soil and prevent its transport to the shoot.

Gas exchange parameters are often used to determine the overall performance of the plants. Salt stress damages the thylakoid membrane—harboring different photosynthetic pigments—and in turn, it decreases the photosynthetic machinery as the first reaction, followed by a decline of plant growth and crop productivity [2]. Salinity can affect photosynthesis directly or indirectly by decreasing CO_2_ availability caused by diffusion limitations. In the present study, saline conditions suppressed F_v_/F_m_, stomatal conductance, and increased chlorophyll degradation; the untreated plants were the most affected (Figure 3). Under salt stress, photosynthesis is inhibited through either a reduction in stomatal conductance or such non-stomatal factors such as a reduction in chlorophyll pigments to absorb enough light [2]. The observed decrease in pigments in untreated plants may be attributed not only to an increased degradation but also to an inhibited synthesis of chlorophylls due to salinity. Similar results were found in alfalfa [9], carob [15], and date palm [3]. Stomata play a crucial role in regulating the uptake of CO_2_ for photosynthesis against the loss of water via transpiration. The ability of plants to regulate their stomatal conductance is a vital preservation mechanism that helps to cool leaves, regulate water loss, and uptake CO_2_. Plants inoculated with AMF and rhizobia strain and/or amended by compost showed an efficient photosystem II (an average F_v_/F_m_ of 0.8) and higher stomatal conductance and photosynthetic pigments than the control (Figure 3), which reflects that the use of biofertilizers ameliorates the physiological state of the plants under both saline conditions. These improvements can lead to boost the photosynthesis rate due to better CO_2_ assimilation. The latter may explain the increase in alfalfa growth and the reduction in the inhibitory effect of salinity on the shoot and root biomass, plant height, and total leaf number (Table 2). A plant’s photosynthetic products were also portioned out to support symbiotic microbes’ reproduction. Several studies have illustrated the importance of inoculation by AMF in mitigating the harmful effects of abiotic stress on plant growth through improving photosynthetic capacity, enhancement of plant water status, and antioxidant activity [10,15]. In the vein, Anli et al. [13] postulated that the treatment of organic amendment and AMF prevented the abiotic stress from inhibiting photosynthesis by improving chlorophyll pigment synthesis and stomatal characteristics, including stomatal pore size and density. The present study demonstrated the synergistic effect of compost, AMF, and *RhOL1* on the photosynthetic attributes of alfalfa. The improvement of chlorophyll synthesis is linked directly to the mineral elements absorption, efficiency, and transport; as they are necessary for heme, chlorophyll synthesis, and as co-factors for several critical cellular processes, including the photosynthesis [2]. It is important to note that compost provides essential nutrients, and root beneficial microbes improve the ability of plants to acquire these nutrients. Still, these microbes can modulate plant phytohormones, e.g., cytokinins, ABA, and auxin, which trigger stomatal conductance (and presumably photosynthesis) to gauge their response to salinity [62]. Thus, the triple combination might deliver alfalfa plants with higher nutrient uptake and phytohormones necessary to increase photosynthesis efficiency and plant growth under salt stress. Additionally, the improved water uptake observed after the application of the amendments could help to maintain a larger leaf area and better stomatal conductance and therefore better CO_2_ assimilation. Interestingly, improvement of the antioxidant activity recorded in this work, after that, by these biofertilizers can protect the photosynthetic machinery against the oxidative damages caused by saline stress [1].

Treatments of compost and root-derived microorganisms not only helped host plant growth but also enhanced the defense system and subsequently reduced salinity severity. The studied amendments applied separately are known to induce profound changes in plant metabolism, including osmoprotectants and antioxidants, which are key components of plant tolerance [3]. To elucidate the underlying biochemical aspects of the observed tolerance in treated plants, the antioxidative system was analyzed (Figure 4 and Figure 5). Salinity induces plants to synthesize/accumulate organic solutes—many of these are N-containing solutes—in their cells, which reduces both osmotic and leaf water potentials; therefore, more water could enter into the cell [63]. Proline is a soluble amino acid whose accumulation in the cytosol under abiotic stress can play a role as an osmoprotectant and osmotic adjustment [64]. In our investigation, salinity enhanced the proline content in alfalfa plants (Figure 5). However, biofertilizers application also further increased the content of proline in alfalfa plants under saline conditions. Enhancement in proline contents by biofertilizers under salinity may be due to a reduced exploitation of proline under salinity stress either because of the low degradation of proline [65] or increased proline synthesis [1] due to the up-regulation of proline biosynthesis gene (P5CS) activity, which is reported to be strongly induced under high salinity [66]. The overproduction and/or accumulation of proline in salt-stressed plants plays an essential role in NaCl tolerance by reducing oxidative stresses caused by ROS [64,66], modulating redox homeostasis, the maintenance of cytosolic pH, protecting photosynthetic apparatus, and stabilizing cellular structures such as membranes and protein through participating in the stress signal [64,67]. Additionally, in the presence of biofertilizers, this metabolite might act as an osmoregulator and osmoprotector; it functions as an osmolyte to decrease leaf water potential, maximizing water uptake, and/or reducing transpiration to maintain cell turgor pressure during conditions associated with the saline condition [64]. In addition to these roles, proline may act as an N source in the cell at the lifting of the saline conditions, where the accumulation of this N-rich compound could be utilized as a form of N storage and energy utilization [68].

Under salinity stress, plants activate an array of antioxidant enzymes such as SOD, CAT, and APX to reduce the harmful effects of stress [67]. This investigation showed an increase in SOD, CAT, and APX activities in alfalfa plants under saline conditions (Figure 5). The addition of biofertilizers improved the activities of SOD, CAT, and APX in salt-stressed alfalfa plants. The application of AMF and/or compost and/or rhizobium triggers particular antioxidant genes, which enhance the activities of enzymatic antioxidants [1,20,69,70]. Therefore, these biofertilizers may be included in the scavenging of ROS species under saline conditions. It is considered that SOD acts “in the first line” of defense against oxidative stress in plants. A further instance of this is that SOD activity and salinity have been reported in glycophytes, where an increase in SOD-specific activity is positively correlated with increasing NaCl concentrations [71]. The produced H_2_O_2_ is dismutated into H_2_O and O_2_ by CAT, which, similar to our data, has been reported to be up-regulated in several plant species [72]. APX plays an essential role in ROS scavenging during salinity and shows a higher affinity for H_2_O_2_ than CAT. APX was found to be activated under salt stress, being higher in several halophytes compared to glycophytic plants [69]. Evidently, phytohormones production (e.g., auxin, cytokinin, ethylene, jasmonic acid, and gibberellins), their signaling pathways, and their interactions are proven to be critical in regulating plant antioxidant defenses and their associated microbiota [73]. In this study, a general pattern of variation in the specific activities of these antioxidant enzymes in response to salt has been observed. The addition of biofertilizers showed higher specific activities for the three enzymes tested under saline conditions, as compared with the untreated controls, and a strong positive correlation—confirmed by the corresponding PCA in (Figure 7)—between salinity and SOD, CAT, and APX. Notably, this pattern of activation of these enzymes as a specific response to salinity agrees with the lower degree of oxidative stress in alfalfa, which was revealed by the lower salt-induced H_2_O_2_, MDA, and EL levels (Figure 4).

Persistent salinity-induced ROS accumulation—resulting from an imbalance between ROS production and its removal by enzymatic and non-enzymatic detoxification mechanisms—causes oxidative stress-associated injuries, including lipid peroxidation measured as MDA and membrane leakage. In our investigation, salt stress enhanced the contents of MDA, EL, and H_2_O_2_ in alfalfa plants. These data are in line with several plants, including alfalfa [9,21] and date palm [3,10]. Under stressful conditions, the excessive accumulation of energy due to the reduction in photosynthetic performance and absorption of more light energy to be consumed by C fixation leads to a generation of ROS in plastids [67]. Further, saline conditions induce EL mainly by K^+^ efflux [74]. In our study, salinity enhanced EL in alfalfa plants. Contrastingly, biofertilizers treatments, single or combined, decreased the contents of H_2_O_2_, MDA, and EL in salinity-stressed alfalfa plants, which is similar to the finding in *Medicago sativa* [9]. This reduction in the concentration of these injuries is due to the increased activity of enzymatic antioxidants and scavenging genes induced by microbes [75] and/or compost [20]. Previous reports have shown that the strength of the cell membrane is enhanced by proteins, which helps in reducing the unsaturation of membranes [76]. These findings were further confirmed by our data. In the present study, the application of composts together with inoculation of the native AMF and rhizobial strains inoculations either as single or combined treatments were very effective in helping alfalfa plants to boost the protein concentration in leaves as compared to compost- and microbes-free controls (Figure 5A). These results are in agreement with previous reports using AMF or PGPR [13].

To visualize the differences among treatments with reference to growth parameters, mineral nutrients, and patterns of changes in defense mechanisms parameters, a PCA analysis was performed. The first PCA axis, which explained 55% of the variation, was positively correlated (right side) with minerals solutes, plant growth (shoot and root weights and leaf number), and photosynthetic parameters. These variables showed their more robust association with plants grown under normal condition and treated with biofertilizers (compost, AMF, and/or rhizobium) treatments. In contrast, the non-inoculated and non-treated control treatments under no-saline and saline conditions, Ct and SS, respectively, were separated into a distinct group (left side) and showed a negative correlation with the measured variables; the stressed plants more vulnerable to salinity. The third group corresponding to other treatments, treated alfalfa under salinity, occurred in intermediate positions and correlated with enzymatic and non-enzymatic antioxidant defenses. These findings showed that the application of compost–AMF–rhizobium is a factor driving to improve the growth and cultivation of alfalfa under both normal and salt-stressed conditions.

**Figure 8:**
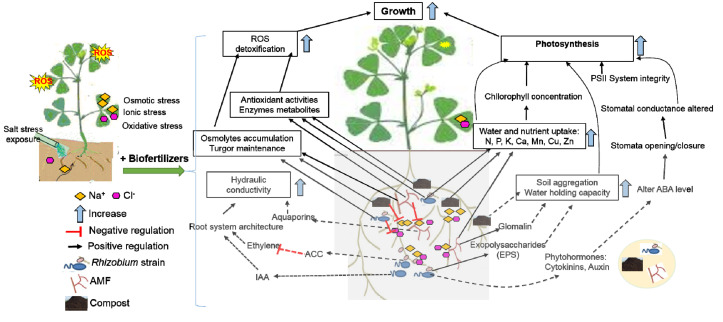
Diagram of signaling cascades induced by compost, rhizobium strain, and arbuscular mycorrhizal fungi leading to salt tolerance in alfalfa plant. Solid lines represent the analyses carried out in this study. Dashed lines indicate mechanisms found in the literature.

Overall, unlike several studies that often include individual “known” beneficial agents, which may underestimate the interactions and signaling between microorganisms and amendments’ supply, this study took into account broader-scale plant microorganisms, including selective native AMF and salt-tolerant rhizobia, and soil amendment with green compost under abiotic stress as a system-based approach in shaping the soil to better understand the plant tolerance and microbial symbionts changes. This would bring us closer to the holy grail in reaching sustainable agriculture under environmental changes. In addition to the below-ground alfalfa-associated microorganisms’ traits, the plant physiology and antioxidant system were integrated in order to identify the system-based response to normal and salt stress conditions. The treatment involving green compost, AMF, and rhizobia strain deems effective, as compared with individual or dual treatments, in improving and preserving alfalfa’s physiological and biochemical traits under salt stress conditions. A possible mechanisms scheme of compost, rhizobia strain, and AMF for inducing salt tolerance in alfalfa by modulating dynamics tolerance expression is presented in Figure 8. The direct mechanisms of biofertilizers include osmolytes accumulation, antioxidant system activation, elevated levels of ROS neutralization, nitrogen fixation, selective uptake of ions, photosynthesis machinery protection, and production of glomalin and EPS-binding cations. These mechanisms lead to increase root length, surface area and root number, and thereby nutrient uptake [3,10]. The indirect mechanism might include, at least partially, phytohormones, ACC deaminase production, and/or systemic tolerance induction, which counteract on salinity stress and maintain cell turgor and stimulate plant growth. Considering such a current scenario, future research is needed to identify potential stress targets at transcriptional, proteomics, and hormonomics levels.

## 5. Conclusions

The results herein hold promise for developing tailor-made systems to improve plant performance under normal and stressed conditions by modifying root-associated bacteria and fungi. Similarly, these microorganisms induce enhanced stress resistance of alfalfa, which is triggered by a set of mechanisms and metabolites that may then work to mitigate plant salt-stress. It should be noted that the tri-combination CMR mitigated on a large scale the salinity-induced negative impact and improved alfalfa growth by enhancing the chlorophyll synthesis, photosynthesis machinery, and antioxidant defense system. Furthermore, the synergy between AMF, *Rhizobium*, and compost improved the uptake of water and nutrients needed by the plants and, most importantly, it induced the secretion of GRSP, which has several beneficial effects in aggregate stability and soil health. The findings here suggest a potential for targeted rhizosphere microbiome engineering to promote plant performance, and as a candidate to support useful plant probiotic tools against stresses, through the application of crucial microbiome.

*Rhizobium* alters root characteristics to facilitate the uptake of water and minerals (N) through hormones such as IAA (indole acetic acid) who increases the root system architecture. ACC deaminase (1-aminocyclopropane-1-carboxylate) reduces the ethylene level to eliminate the negative effect on roots. ABA mediate stomatal opening and closure and help maintain photosystem functionality under salt stress. The production of osmoprotectants by rhizobia reduces the damaging effect of ROS and also contributes toward salt stress tolerance. Furthermore, soil aggregation due to the production of EPS helps in increased water-holding capacity and limits the content of salt (Na^+^ and Cl^−^) available to roots. AMF colonization of a plant root produced glomalin and allowed the extension of hyphae, providing availability and the storage of nutrients for the plant. In addition, the synthesis of aquaporins by AMF can enhance water uptake. AMF can also limit the availability of salt by storing it in their vacuole. Compost improves nutrients uptake, soil aggregation, and water-holding capacity through the activity of soil microorganisms, which provide the optimum environment of other soil microorganisms such as AMF and rhizobia. Therefore, the combined use of compost–rhizobia–AMF regulates physiological and biochemical processes and improves salt-stress tolerance of alfalfa.

## Figures and Tables

**Table 1:** Characteristics of rhizobium strain (*RhOL1*).

Phosphate Solubilization (mg L^−1^)	Potassium Solubilization (HD/CD)	Production of Exopolysaccharide (EPS) (mg of CR/OD_600_)	Production of IAA (µg mL^−1^)	Nitrogen Fixation
1.55 ± 0.01	2.19 ± 0.06	156.57 ± 10.60	32.37 ± 0.22	+

HD: halo diameter; CD: colony diameter; CR: Congo Red; OD: optical density; IAA: indole-3-acetic acid; +**:** presence of nitrogen fixation. Values are means ± SE.

**Table 2:** Arbuscular mycorrhizal fungi (AMF) infection frequency and intensity, nodules dry weight, plant height, leaf number, and leaf water content of alfalfa plants grown without (0 mM NaCl) or with (120 mM NaCl) salt stress and subjected to different biofertilizers treatments; Ct: untreated control, M: inoculated with arbuscular mycorrhizal fungi, R: inoculated with rhizobium strain, C: amended with compost, MR: inoculated with the mixture AMF+rhizobium, CM: amended with compost and inoculated with the AMF, CR: amended with compost and inoculated with rhizobium, and CMR: amended with compost and inoculated with the mixture AMF+ rhizobium.

	AMF Infection Frequency (%)		AMF Infection Intensity (%)		Nodules Dry Weight (mg)		Plant Height (cm)		Root Length (cm)		Leaf Number		Leaf Water Content (%)	
	0 mM	120 mM	0 mM	120 mM	0 mM	120 mM	0 mM	120 mM	0 mM	120 mM	0 mM	120 mM	0 mM	120 mM
Ct	-	-	-	-	-	-	19.7 ± 0.8 ^g^	13.3 ± 2.2 ^h^	18.6 ± 0.3 ^e^	14.0 ± 0.5 ^d^	9.5 ± 0.3 ^hi^	6.0 ± 0.6 ^i^	59.7 ± 1.7 ^g^	48.9 ± 1.3 ^h^
M	86.6 ± 3.3 ^a^	66.6 ± 3.3 ^b^	25.7 ± 5.0 ^b^	7.0 ± 2.2 ^c^	-	-	31.5 ± 0.3 ^ef^	20.1 ± 0.8 ^g^	24.0 ± 0.5 ^b^	19.3 ± 0.6 ^c^	18.0 ± 0.4 ^fgh^	15.2 ± 1.9 ^ghi^	71.0 ± 5.0 ^def^	63.7 ± 3.1 ^fg^
R	-	-	-	-	80.0 ± 0.0 ^e^	60.0 ± 0.0 ^f^	33.7 ± 1.5 ^de^	20.1 ± 1.1 ^g^	24.3 ± 0.3 ^b^	19.6 ± 0.6 ^c^	24.5 ± 0.5 ^defg^	16.2 ± 2.2 ^fgh^	71.7 ± 0.2 ^cdef^	64.9 ± 2.0 ^fg^
MR	85.0 ± 5.0 ^a^	69.6 ± 2.0 ^b^	38.0 ± 1.0 ^a^	6.7 ± 2.8 ^c^	80.0 ± 0.0 ^e^	60.0 ± 0.0 ^f^	36.3 ± 2.1 ^cd^	23.0 ± 1.4 ^g^	25.0 ± 1.5 ^b^	20.3 ± 0.5 ^c^	32.2 ± 0.7 ^cde^	26.2 ± 2.1 ^cdef^	77.9 ± 5.6 ^bcd^	69.1 ± 2.4 ^ef^
C	33.3 ± 4.0 ^e^	6.6 ± 0.0 ^g^	0.6 ± 0.1 ^e^	0.4 ± 0.2 ^e^	30.0 ± 0.0 ^g^	15.0 ± 0.0 ^i^	39.7 ± 2.7 ^bc^	21.7 ± 1.7 ^g^	27.1 ± 2.5 ^b^	19.0 ± 0.6 ^c^	29.5 ± 1.9 ^cde^	22.5 ± 1.5 ^efg^	76.1 ± 1.4 ^bcd^	70.9 ± 2.6 ^def^
CM	40.0 ± 5.7 ^d^	31.0 ± 1.0 ^e^	5.6 ± 3.1 ^c^	2.0 ± 1.3 ^d^	30.0 ± 0.0 ^g^	20.0 ± 0.0 ^h^	42.6 ± 1.7 ^b^	31.2 ± 0.8 ^ef^	33.3 ± 0.6 ^a^	20.6 ± 0.3 ^c^	52.5 ± 4.2 ^a^	33.0 ± 1.8 ^cd^	82.1 ± 1.9 ^ab^	75.6 ± 2.2 ^bcde^
CR	36.3 ± 1.9 ^d^	16.5 ± 1.8 ^fg^	0.8 ± 0.1 ^e^	0.4 ± 0.1 ^e^	130.0 ± 0.0 ^b^	90.0 ± 0.0 ^d^	40.2 ± 1.2 ^bc^	28.0 ± 0.8 ^f^	26.3 ± 1.0 ^b^	18.6 ± 0.3 ^c^	44.0 ± 3.0 ^ab^	34.2 ± 3.4 ^bcd^	80.0 ± 0.7 ^bc^	76.1 ± 2.7 ^bcde^
CMR	56.5 ± 2.0 ^c^	38.0 ± 2.8 ^d^	5.0 ± 2.2 ^c^	0.5 ± 0.4 ^e^	140.0 ± 0.0 ^a^	120.0 ± 0.0 ^c^	51.0 ± 1.5 ^a^	33.5 ± 2.1 ^de^	32.3 ± 1.4 ^a^	24.0 ± 1.4 ^b^	51.0 ± 2.9 ^a^	35.5 ± 3.4 ^bc^	89.3 ± 0.9 ^a^	79.3 ± 1.3 ^bcd^

Means (± standard error) sharing similar letters within the same parameter are statistically non-significant at *p* < 0.05 (Tukey’s test).

## Supplementary Materials

The supplementary material for this article can be found online at https://www.mdpi.com/2076-2607/8/11/1695/s1. Table S1: Result of multivariate analysis of variance MANOVA test for independent variables including salt stress treatments (SS; 0 mM and 120 mM), arbuscular mycorrhizal fungi (M) and rhizobium bacteria (R) inoculations, compost (C) amendment, and the interaction among them. Table S2: Loading values and percentage contribution of variables on the axis identified by the principal component analysis for all treatments under saline and non-saline conditions.

## Abbreviations

ABA ACC	abscisic acid 1-aminocyclopropane-1-carboxylate
AMF	Arbuscular Mycorrhizal Fungi
APX	ascorbate peroxidase activity
CAT	catalase activity
Chl	total chlorophyll content
EC	electrical conductivity
EE-GPRS	easily extractable glomalin-related soil protein
EL	electrolyte leakage
EPS	exopolysaccharides
Fa	AMF infection frequency
F_v_/F_m_	chlorophyll fluorescence
IAA	indole acetic acid
g_s_	stomatal conductance
H_2_O_2_	hydrogen peroxide content
LWC	leaf water content
Ma	AMF infection intensity
MDA	malondialdehyde content
NDW	nodule dry weight
NL	leaf number
PH	plant height
RDW	root dry weight
RL	root length
ROS	reactive oxygen species
SDW	shoot dry weight
SOD	superoxide dismutase activity
T-GPRS	total extractable glomalin-related soil protein
