# Supplementary material for: Potential of Native Arbuscular Mycorrhizal Fungi, Rhizobia, and/or Green Compost as Alfalfa (Medicago sativa) Enhancers under Salinity
Source: Microorganisms. 2020 Oct 30;8(11):1695. doi: 10.3390/microorganisms8111695 (PMC7693256; doi:10.3390/microorganisms8111695)
Supplement: Supplementary file 1 [file microorganisms-08-01695-s001.pdf]

Table S1: Result of multivariate analysis of variance MANOVA test for independent variables including salt stress treatments (SS; 0 mM and 120 mM), arbuscular mychorrhizal fungi (M) and rhizobium bacteria (R) inoculations, compost (C) amendment, and the interaction among them.

Parameters	SS	M	R	C	SS*M	SS*R	SS*C	M*R	M*C	R*C	SS*M*R	SS*M*C	SS*R*C	M*R*C	SS*M*R*C
SDW	***	***	***	***	*	**	***	ns	***	ns	ns	**	ns	ns	ns
RDW	***	***	***	***	ns	ns	***	ns	ns	ns	*	ns	*	**	ns
PH	***	***	***	***	*	**	ns	ns	ns	ns	ns	ns	ns	*	ns
RL	***	***	**	***	ns	ns	***	ns	ns	**	ns	ns	ns	**	ns
NL	***	***	***	***	*	ns	**	**	ns	*	ns	*	ns	ns	ns
Fa	***	***	*	***	***	**	***	*	***	**	**	***	*	*	*
Ma	***	***	*	***	***	**	***	*	***	**	**	***	*	*	*
NDW	***	ns	***	***	ns	*	ns	ns	ns	***	ns	ns	ns	ns	ns
LWC	***	***	***	***	ns	ns	ns	ns	ns	ns	ns	ns	ns	ns	ns
P	***	***	***	***	**	***	ns	***	***	***	***	**	ns	***	ns
N	***	***	***	***	ns	ns	*	ns	ns	ns	***	ns	ns	ns	ns
Na	***	***	***	***	***	*	***	***	ns	***	***	ns	***	**	ns
Cl	***	***	***	ns	***	***	*	ns	*	*	ns	ns	*	*	ns
K	***	***	***	***	***	***	***	***	***	*	ns	***	***	***	***
Ca	***	***	***	***	ns	***	***	ns	***	ns	**	ns	ns	***	*
Fv/Fm	***	***	***	***	ns	**	***	***	***	***	ns	ns	**	***	ns
gs	***	***	***	***	ns	*	***	*	***	***	ns	*	*	ns	ns
Chl	***	**	***	**	*	*	***	ns	*	ns	ns	ns	ns	ns	*
EL	***	ns	ns	*	ns	*	*	ns	**	**	ns	ns	ns	ns	ns
MDA	***	***	***	***	***	***	***	***	***	***	***	***	***	***	***
H_2O_2	***	***	***	***	***	***	***	***	***	***	***	***	***	***	***
Proline	***	**	ns	ns	ns	ns	*	**	ns	ns	ns	ns	ns	**	ns
Protein	***	***	***	***	**	ns	***	ns	ns	**	ns	ns	**	**	ns
SOD	***	***	***	***	*	*	*	ns	ns	ns	ns	ns	ns	ns	ns
CAT	ns	***	***	***	s	ns	ns	***	**	ns	ns	ns	ns	ns	ns
APX	***	ns	ns	**	*	ns	**	ns	ns	ns	ns	ns	ns	ns	ns
EE-GPRS	**	***	**	***	ns	ns	*	*	ns	ns	ns	*	ns	ns	ns
T-GPRS	***	***	**	***	ns	ns	ns	*	ns	ns	ns	ns	ns	ns	ns
EC	***	**	***	***	***	***	***	***	ns	***	**	ns	*	***	***

ns, not significant, *$p < 0.05$, **$p < 0.01$, ***$p < 0.001$.

SDW: shoot dry weight, **RDW**: root dry weight, **PH**: plant height, **RL**: roots length, **NL**: leaf number, **Fa**: AMF infection frequency, **Ma**: AMF infection intensity, **NDW**: nodule dry weight, **LWC**: leaf water content, **P**: phosphorus uptake, **N**: nitrogen uptake, **Na**: sodium uptake, **Cl**: chlore uptake, **K**: potassium uptake, **Ca**: calcium uptake, **F_v/F_m**: chlorophyll fluorescence, **g_s**: stomatal conductance, **Chl**: total chlorophyll content , **EL**: electrolyte leakage, **MDA**: malondialdehyde content, **H_2O_2**: hydrogen peroxide content, **Proline**: proline content, **Protein**: protein content, **SOD**: superoxide dismutase content, **CAT**: catalase content, **APX**: ascorbate peroxidase content, **EE-GPRS**: easily extractable glomalin-related soil protien, **T-GPRS**: Total extractable glomalin-related soil protien, **EC**: electrical conductivity.

Table S2. Loading values and percent contribution of variables on the axis identified by the principal component analysis for all treatments under saline and non-saline conditions

Variable	PC1		PC2		PC3	
	Loading values	Contribution of variable (%)	Loading values	Contribution of variable (%)	Loading values	Contribution of variable (%)
SDW	**0.899**	5.045	0.198	0.619	-0.323	4.772
RDW	**0.911**	5.175	0.184	0.538	-0.278	3.550
PH	**0.973**	5.906	-0.064	0.064	-0.129	0.763
RL	**0.929**	5.380	-0.172	0.469	-0.088	0.356
NL	**0.908**	5.142	0.261	1.077	-0.203	1.886
Fa	0.426	1.133	-0.126	0.251	**0.568**	14.766
Ma	0.242	0.367	-0.443	3.114	**0.525**	12.600
NDW	**0.648**	2.617	0.287	1.307	-0.223	2.286
LWC	**0.961**	5.045	0.239	0.619	0.051	4.772
P	**0.841**	4.409	0.406	2.614	-0.101	0.470
N	**0.909**	5.156	-0.013	0.003	-0.196	1.764
Na	**-0.520**	1.685	**0.766**	9.308	-0.216	2.140
Cl	**-0.732**	3.344	**0.552**	4.841	-0.253	2.922
K	**0.914**	5.210	0.114	0.205	-0.279	3.555
Ca	**0.913**	5.198	-0.015	0.003	0.010	0.004
Fv/Fm	**0.835**	4.348	0.097	0.150	0.370	6.254
gs	**0.898**	5.031	0.029	0.013	0.022	0.022
Chl	**0.808**	4.073	-0.395	2.473	-0.138	0.867
EL	-**0.787**	3.862	0.363	2.085	-0.352	5.686
MDA	**-0.751**	3.520	-0.011	0.002	-0.458	9.596
H_2O_2	**-0.743**	3.444	-0.039	0.024	-0.523	12.544
Proline	-0.302	0.568	0.693	7.626	0.294	3.960
Protein	0.909	5.150	-0.156	0.385	-0.124	0.700
SOD	-0.088	0.048	0.926	13.600	0.229	2.401
CAT	0.089	0.049	0.952	14.374	0.146	0.982
APX	-0.158	0.155	**0.884**	12.404	0.306	4.284
EE-GPRS	**0.809**	4.087	0.493	3.862	-0.054	0.134
T-GPRS	**0.809**	4.087	0.493	3.862	-0.054	0.134
EC	-0.090	0.051	0.933	13.818	0.102	0.480
Eigenvalue	16.032		6.305		2.184	
Variability (%)	55.284		21.741		7.531	
Cumulative %	55.284		77.025		84.555	

SDW: shoot dry weight, **RDW**: root dry weight, **PH**: plant height, **RL**: roots length, **NL**: leaf number, **Fa**: AMF infection frequency, **Ma**: AMF infection intensity, **NDW**: nodule dry weight, **LWC**: leaf water content, **P**: phosphorus, **N**: nitrogen, **Na**: sodium, **Cl**: chlore, **K**: potassium, **Ca**: calcium, **F_v/F_m**: chlorophyll fluorescence, **g_s**: stomatal conductance, **Chl**: total chlorophyll content , **EL**: electrolyte leakage, **MDA**: malondialdehyde content, **H_2O_2**: hydrogen peroxide content, **Proline**: proline content, **Protein**: protein content, **SOD**: superoxide dismutase content, **CAT**: catalase content, **APX**: ascorbate peroxidase content, **EE-GPRS**: easily extractable glomalin-related soil protien, **T-GPRS**: Total extractable glomalin-related soil protien, **EC**: electrical conductivity.

Values in bold represented >50% contribution to the significant component.
